# Supplementary material for: Time-varying effects are common in genetic control of gestational duration
Source: Hum Mol Genet. 2023 May 17;32(14):2399–407. doi: 10.1093/hmg/ddad086 (PMC10321382; doi:10.1093/hmg/ddad086)
Supplement: suppl_material_ddad086 [file suppl_material_ddad086.pdf]

Supplementary material

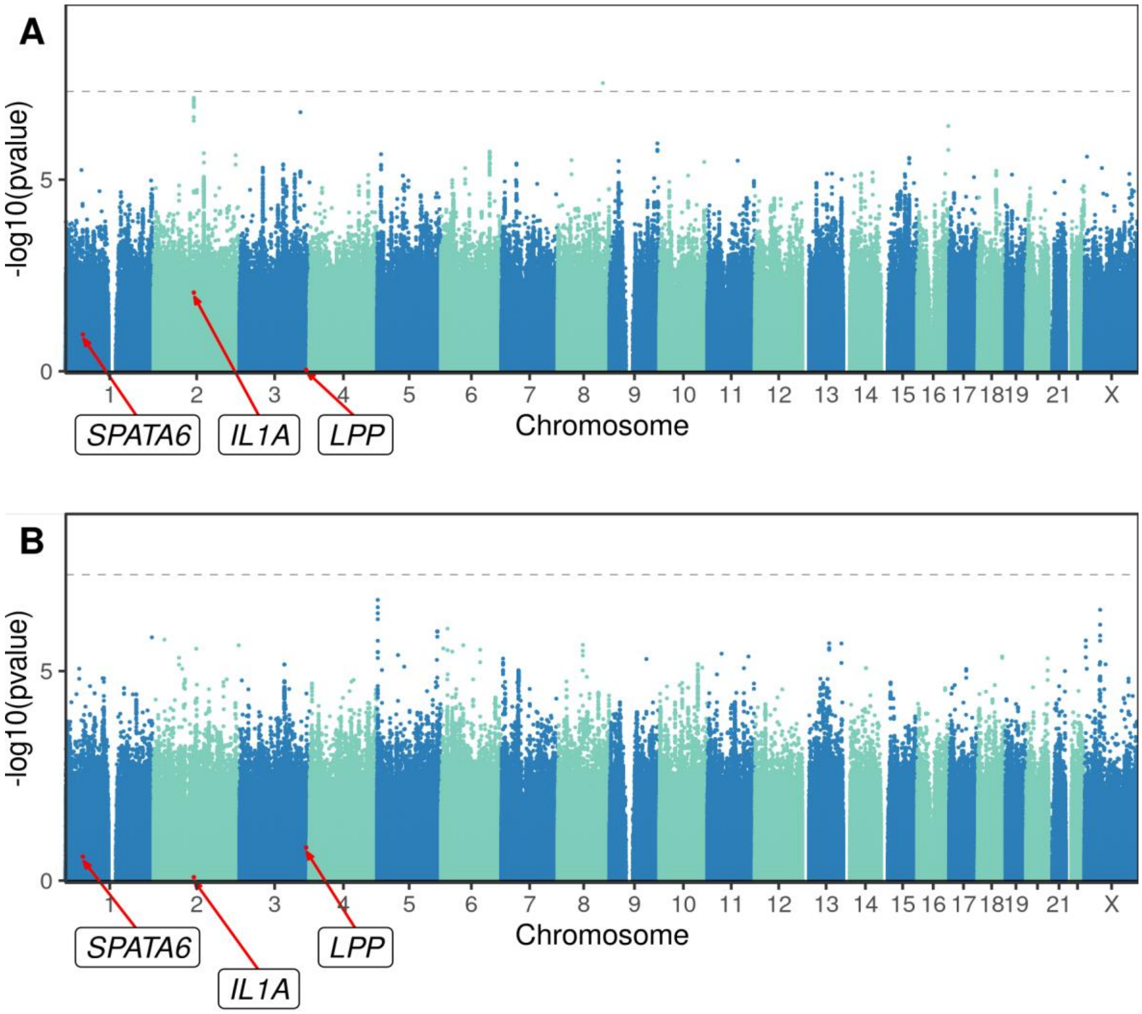

Figure S1. Manhattan plot illustrating the foetal GWAS results for gestational duration (A) and preterm delivery (B). The dashed line marks the significance threshold. Note the one genome-wide significant association for gestational duration in chromosome 8. No genome-wide significant association in the foetal GWAS of preterm delivery was detected. The variants marked in red, and the corresponding loci, were reported as significant in the largest previous foetal GWAS meta-analysis of gestational timing (Liu et al. 2019).

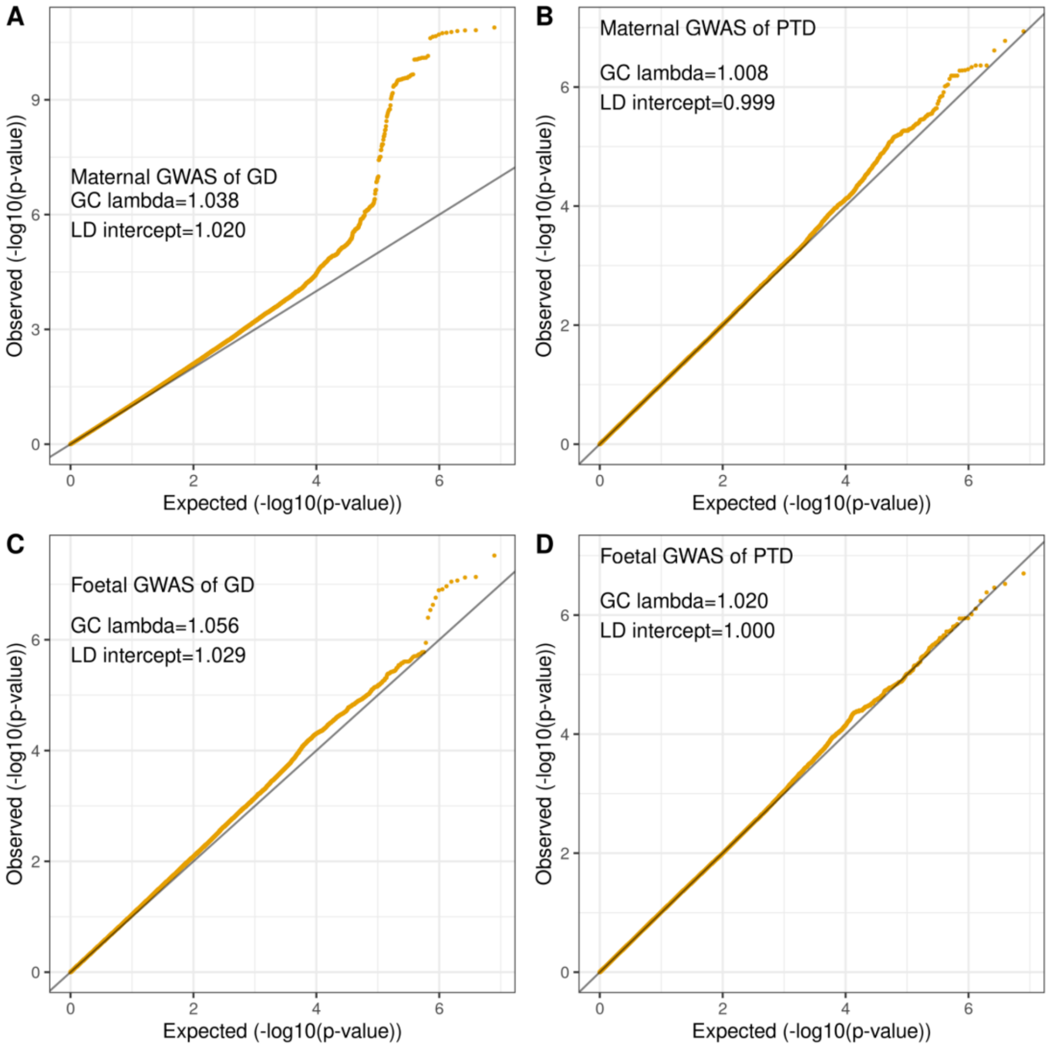

Figure S2. Quantile-quantile plots of maternal (A,B) or foetal (C,D) GWASs of gestational duration (A,C) and preterm delivery (B,D). Lambda genomic control and LD score regression intercept values are shown in the labels, and show little to none test statistic inflation in all cases.

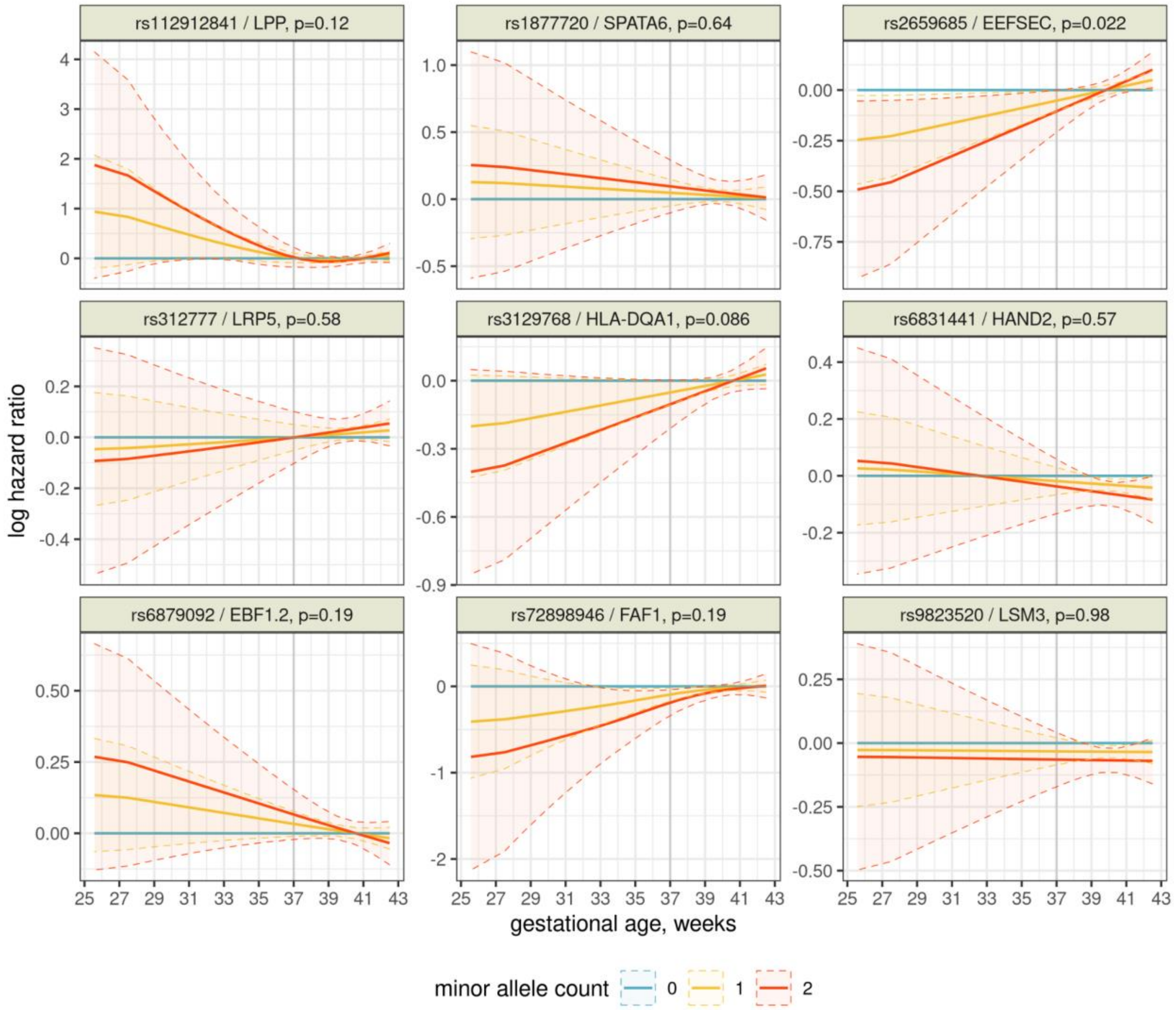

Figure S3. Effects of 9 genetic variants on delivery timing throughout gestation, estimated by a flexible PAMM. Variants shown here were detected in a previous meta-analysis, but had no significant effects in the GWAS in this cohort. For each week of gestation, the line is the estimated instantaneous log hazard ratio (positive=risk of birth is currently increased) and its 95 % confidence interval is shaded. *EBF1.2* is a second association reported near the *EBF1* gene. Note that the minor allele count was used as a continuous covariate in the analysis, so the effects of 1 and 2 minor alleles are constrained to be proportional.

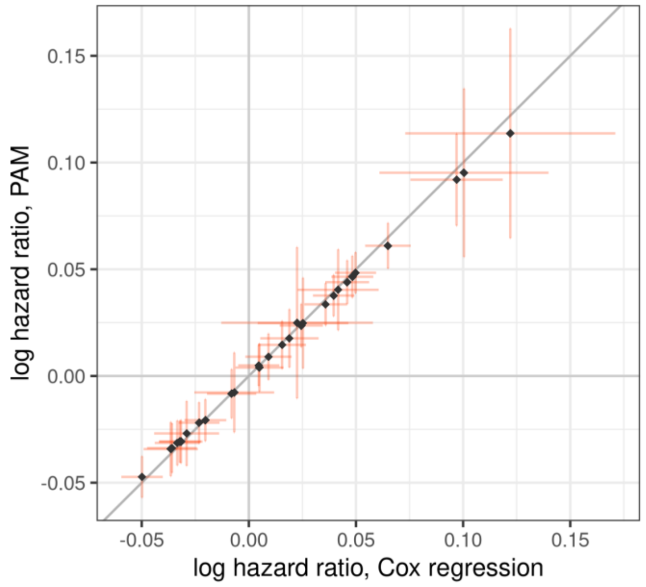

Figure S4. Comparison between the variant effect estimates from Cox regression and a simplified PAM with only constant (parametric) effects. Points are the estimates and bars are +/- 1 SE. The diagonal grid line representing equal effects is highlighted.

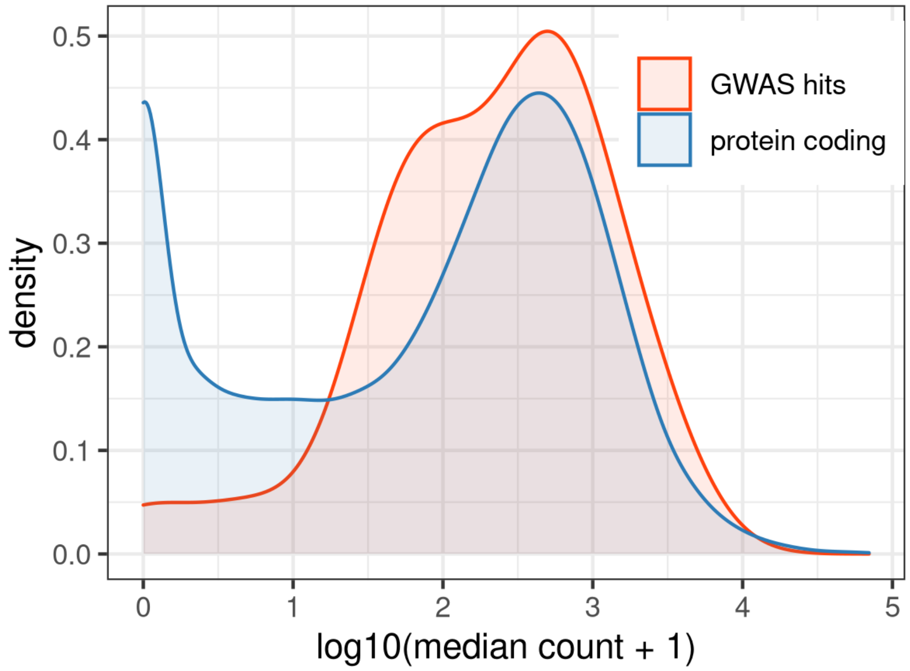

Figure S5. Distribution of median read counts for different genes in placental expression data (median taken over samples). “GWAS hits” are the 28 distinct genes corresponding to the loci analysed in detail in this study, “protein coding” are all other protein coding genes identified in the Ensembl database.

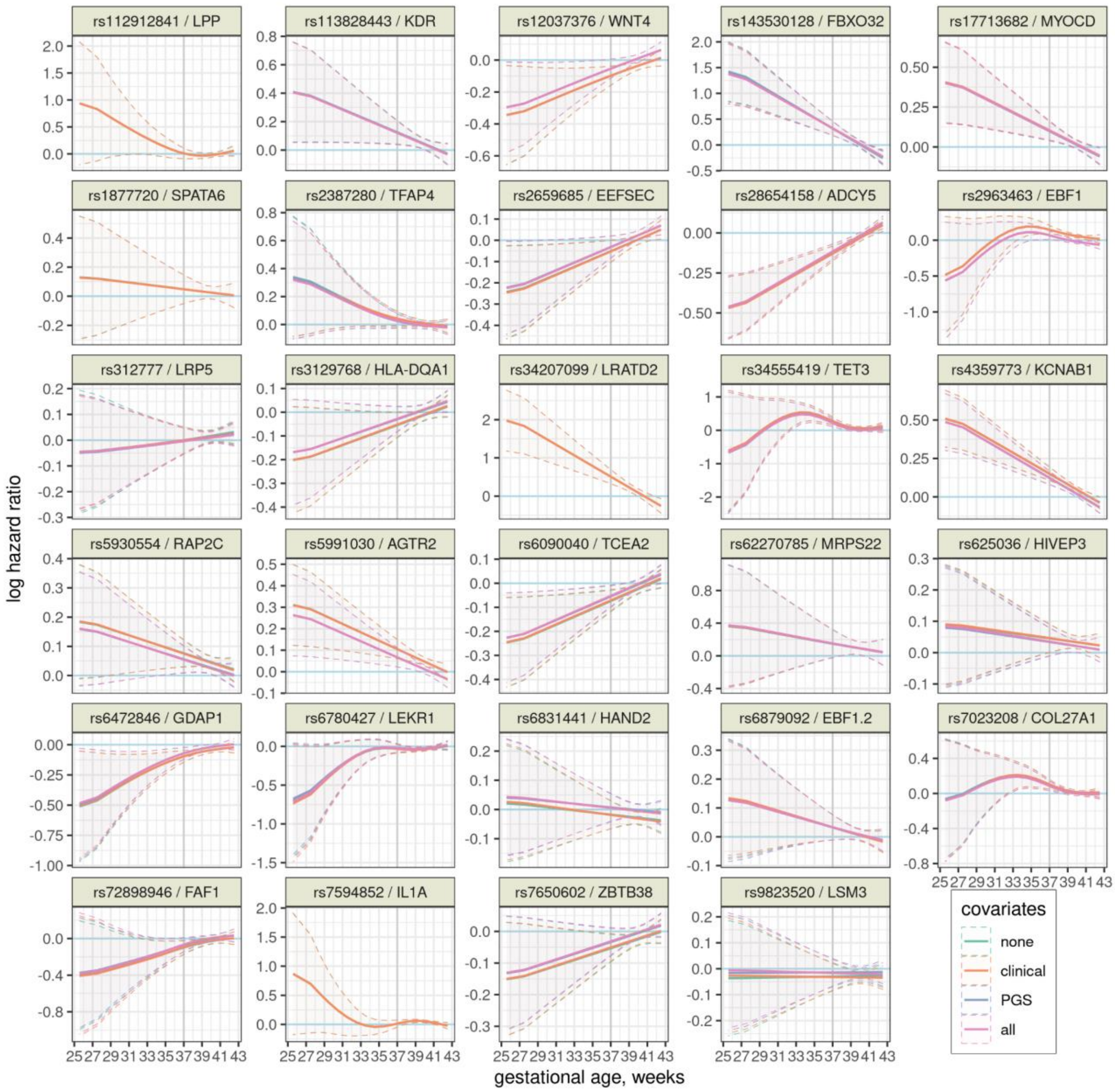

Figure S6. Effects of 29 genetic variants on delivery timing throughout gestation, estimated by flexible PAMMs with different covariates. The effects are shown per one copy of minor allele. For each week of gestation, the line is the estimated instantaneous log hazard ratio (positive=risk of birth is currently increased) and its 95 % confidence interval is shaded. PGS: maternal polygenic score, only shown for the maternal loci. Note that the lines with or without clinical covariates overlap almost perfectly.

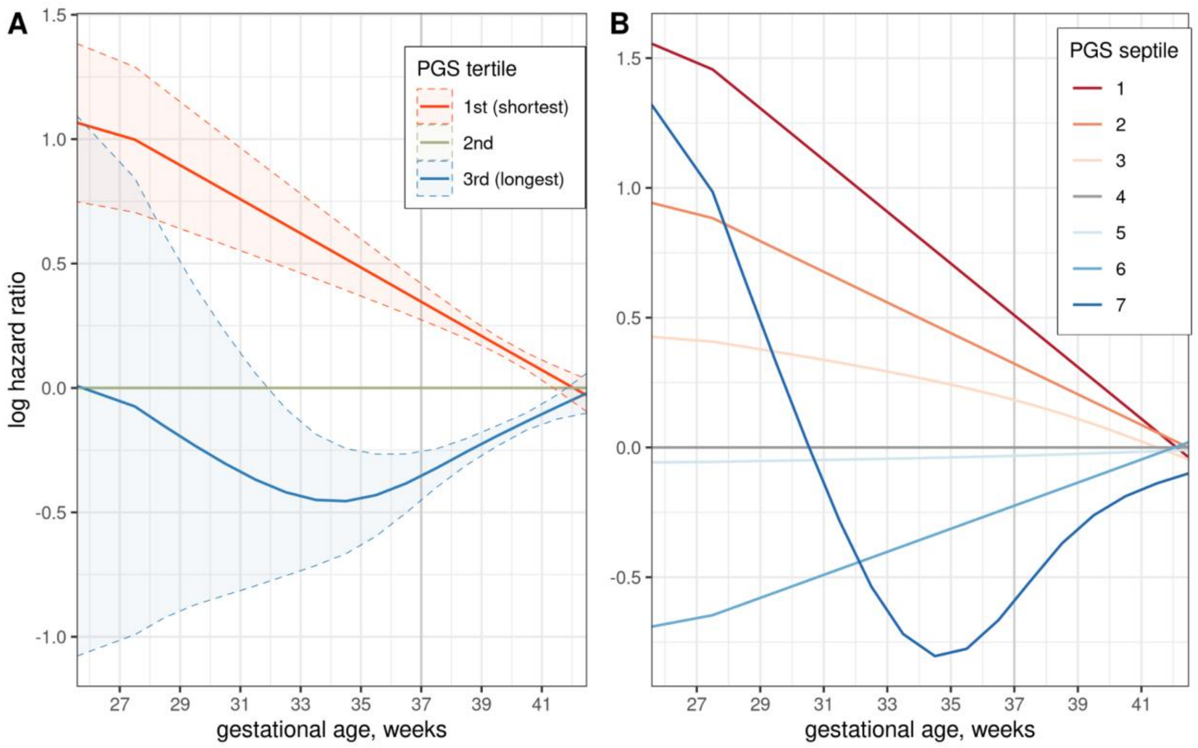

Figure S7. Effects of the gestational duration polygenic score throughout gestation, estimated by a flexible PAMM. The polygenic score is split into 3 (A) or 7 (B) categories. For each week of gestation, the line is the estimated instantaneous log hazard ratio (positive=risk of birth is currently increased). The 95 % confidence intervals are shaded in the 3-group plot, but for clarity not shown in the 7-group plot.

locus	rsid	chr	pos	ref	eff	EAF	lin.beta	pc.sm.p	pc.int.p	cox.sch.p
KCNAB1	rs4359773	3	155862524	A	G	0.43	-0.65	1.2e-06	2.7e-07	7.1e-05
FBXO32	rs143530128	8	124603784	G	A	0.02	-1.5	4.1e-06	2.5e-06	0.00039
LRATD2	rs34207099	8	127494476	A	T	0.014	-2.7	4.3e-06	3.1e-06	0.00036
ADCY5	rs28654158	3	123112292	T	C	0.38	0.52	7.3e-06	4.6e-06	1.1e-05
MYOCD	rs17713682	17	12416363	G	T	0.19	-0.4	0.0023	0.0019	0.011
TET3	rs34555419	2	74235926	A	G	0.055	-1	0.0033	0.015	0.097
AGTR2	rs5991030	X	115129904	T	C	0.43	-0.48	0.0053	0.0043	0.017
EBF1	rs2963463	5	157895049	C	T	0.27	-0.55	0.011	0.01	0.0091
TCEA2	rs6090040	20	62692060	A	C	0.46	0.28	0.016	0.014	0.0058
COL27A1	rs7023208	9	116935764	C	G	0.34	-0.33	0.019	0.015	0.12
EEFSEC	rs2659685	3	128122396	G	A	0.24	0.2	0.022	0.019	0.0067
GDAP1	rs6472846	8	75315146	G	C	0.41	0.48	0.035	0.017	0.095
KDR	rs113828443	4	55895282	C	T	0.075	-0.42	0.039	0.034	0.07
IL1A	rs7594852	2	113521754	C	T	0.46	-0.24	0.042	0.29	0.15
WNT4	rs12037376	1	22462111	G	A	0.17	0.36	0.043	0.021	0.084
HLA-DQA1	rs3129768	6	32595083	T	G	0.22	0.19	0.086	0.074	0.071
LEKR1	rs6780427	3	156697097	A	G	0.4	0.33	0.11	0.14	0.051
LPP	rs112912841	3	187987683	A	G	0.073	0.021	0.12	0.93	0.32
RAP2C	rs5930554	X	131312089	T	C	0.32	-0.37	0.15	0.14	0.23
ZBTB38	rs7650602	3	141147414	T	C	0.47	0.26	0.16	0.15	0.18

TFAP4	rs2387280	16	4339684	A	T	0.27	-0.22	0.18	0.12	0.22
EBF1.2	rs6879092	5	158058432	T	G	0.5	-0.15	0.19	0.17	0.3
FAF1	rs72898946	1	50959262	A	C	0.11	0.28	0.19	0.072	0.42
MRPS22	rs62270785	3	139004333	A	G	0.016	-0.79	0.49	0.48	0.78
HIVEP3	rs625036	1	42270779	G	A	0.36	-0.29	0.55	0.54	0.82
HAND2	rs6831441	4	174733296	T	G	0.31	0.13	0.57	0.56	0.99
LRP5	rs312777	11	68107264	C	G	0.26	-0.034	0.58	0.52	0.62
SPATA6	rs1877720	1	48824407	C	T	0.054	-0.32	0.64	0.62	0.48
LSM3	rs9823520	3	14293832	G	A	0.22	0.21	0.98	0.95	0.88

Table S1. The top variants tested in time-varying models, and comparison of three methods for testing if the effects vary in time. Ref, eff – reference and effect alleles; EAF – effect allele frequency; lin.beta – effect size on gestational duration in days, from linear regression; pc.sm.p, pc.int.p, cox.sch.p are p-values from, respectively, a PAM model with smoothly varying effects, a PAM model with a linear interaction term, and a Schoenfeld residuals test with a Kaplan-Meier transform. Note that *EBF1.2* is used throughout this paper to refer to a second association in the *EBF1* locus.

**Term or pathway**	**all**	**TV**	**early**
positive regulation of macromolecule biosynthetic process	0.00017	0.00022	0.0019
positive regulation of biosynthetic process	0.00039	0.00045	0.0035
Primary ovarian insufficiency	0.046	0.014	0.0041
positive regulation of DNA-templated transcription	0.00043	0.00088	0.01
positive regulation of nucleic acid-templated transcription	0.00043	0.00088	0.01
positive regulation of RNA biosynthetic process	0.00045	0.00091	0.01
positive regulation of RNA metabolic process	0.001	0.0018	0.018
Proteoglycans in cancer	ns	ns	0.025
positive regulation of cellular biosynthetic process	0.0025	0.004	0.034
positive regulation of kidney development	ns	ns	0.036
positive regulation of nucleobase-containing compound metabolic process	0.00033	0.0045	0.037
cellular response to endogenous stimulus	ns	ns	0.042
translation elongation factor activity	ns	ns	0.05
aromatic compound biosynthetic process	0.032	ns	ns
biosynthetic process	0.028	0.01	ns
cellular biosynthetic process	0.049	0.03	ns
cellular nitrogen compound biosynthetic process	0.0083	0.0064	ns
DNA-templated transcription	0.032	ns	ns
heterocycle biosynthetic process	0.031	ns	ns
macromolecule biosynthetic process	0.0071	0.0056	ns
mesenchymal cell proliferation	0.037	ns	ns
nucleic acid-templated transcription	0.033	ns	ns
nucleobase-containing compound biosynthetic process	0.025	0.049	ns
organic substance biosynthetic process	0.023	0.0085	ns
positive regulation of cellular metabolic process	0.0035	0.016	ns

positive regulation of macromolecule metabolic process	0.028	ns	ns
positive regulation of nitrogen compound metabolic process	0.0064	0.027	ns
regulation of biosynthetic process	0.006	0.0086	ns
regulation of cellular biosynthetic process	0.026	ns	ns
regulation of DNA-templated transcription	0.021	ns	ns
regulation of macromolecule biosynthetic process	0.0028	0.0046	ns
regulation of nucleic acid-templated transcription	0.021	ns	ns
regulation of RNA biosynthetic process	0.023	ns	ns
RNA biosynthetic process	0.037	ns	ns

Table S2. Gene sets significantly enriched and the corresponding p-values: among all 28 genes analysed in this study (“all”), among the 21 gene with significant time-varying effects in our cohort (“TV”), or among 17 genes with effects strongest in the preterm range (“early”). “ns” – not significant.
